# Pathologic Response and Survival Outcomes on HER2-Low vs. HER2-Zero in Breast Cancer Receiving Neoadjuvant Chemotherapy

**DOI:** 10.3390/medicina61071168

**Published:** 2025-06-27

**Authors:** Rumeysa Colak, Caner Kapar, Ezgi Degerli, Seher Yildiz Tacar, Aysegul Akdogan Gemici, Nursadan Gergerlioglu, Serdar Altinay, Mesut Yilmaz

**Affiliations:** 1 Department of Medical Oncology, Bakirkoy Dr. Sadi Konuk Training and Research Hospital, Istanbul 34147, Turkey; kaparcaner@gmail.com (C.K.); ezgitastan.19@gmail.com (E.D.); sehertcr@gmail.com (S.Y.T.); mesutyilmaz12@yahoo.com (M.Y.); 2 Department of Radiology, Istanbul Medipol Mega University Hospital, Istanbul 34214, Turkey; aysegulakdogan@yahoo.com; 3 Department of Pathology, Faculty of Medicine, Arel University, Istanbul 34010, Turkey; nursadan@gmail.com; 4 Department of Pathology, Hamidiye Faculty of Medicine, University of Health Sciences, Istanbul 34371, Turkey; drserdara@yahoo.com

**Keywords:** HER2-low, breast cancer, neoadjuvant chemotherapy, pathologic complete response, disease-free survival, hormone receptor status

## Abstract

*Background and Objectives*: The clinical value of HER2-low breast cancer (BC), defined by immunohistochemistry (IHC) scores of 1+ or 2+/ISH-negative without HER2 amplification, remains unclear in the neoadjuvant setting. This study aimed to determine whether HER2-low and HER2-zero tumors differ in pathological complete response (pCR) rates and disease-free survival (DFS) among early-stage breast cancer patients undergoing neoadjuvant chemotherapy (NAC). *Materials and Methods*: We retrospectively analyzed 134 early BC patients treated with NAC between 2017 and 2023. Patients were categorized as HER2-zero (IHC 0) or HER2-low (IHC 1+ or 2+/ISH–). The primary endpoint was total pCR (tpCR); secondary endpoints included breast (bpCR), nodal (npCR), and radiologic complete response (rCR), alongside DFS analysis stratified by hormone receptor (HR) status. *Results*: Of the cohort, 91 patients (67.9%) were HER2-zero and 43 (32.1%) were HER2-low. There was no statistically significant difference in tpCR (26.4% vs. 27.9%, *p* = 0.852), bpCR (28.6% vs. 30.2%, *p* = 0.843), npCR (37.4% vs. 32.6%, *p* = 0.588), and rCR (23.1% vs. 30.2%, *p* = 0.374) between HER2-zero and HER2-low groups. DFS did not significantly differ between HER2-zero and HER2-low groups overall (*p* = 0.714), nor within HR-positive (*p* = 0.540) or TNBC (*p* = 0.523) subgroups. *Conclusions*: HER2-low tumors demonstrated similar pathological responses and survival outcomes compared to HER2-zero tumors. While a HER2-low status does not appear to define a distinct biological subtype in early BC, it remains a relevant classification for emerging HER2-targeted therapies, needing further investigation in prospective studies.

## 1. Introduction

Patients with human epidermal growth factor receptor 2 (HER2)-positive breast cancer (BC) have been the subject of extensive investigation for over 20 years. This subtype accounts for 15–20% of all breast cancer cases, exhibits aggressive biological behavior, and has a poor prognosis [1]. Developing anti-HER2 agents has considerably changed disease progression and improved patient outcomes [2].

Breast cancer is categorized as either HER2-positive or HER2-negative in clinical practice. However, there is significant heterogeneity within HER2 disease, and the current definition of HER2 does not adequately express the clinical and biological variations inherent in HER2 disease [3]. A notable percentage (45–55%) of cancers classified as HER2-negative exhibit low to moderate HER2 expression without HER2 amplification, now termed “HER2-low BC” [4,5].

For individuals in this group, standard HER2-targeted treatments, such as trastuzumab and pertuzumab, offer a slight advantage [6]. Transtuzumab deruxecan (T-DXd) and trastuzumab duocarmazine (SYD985) are two antibody–drug conjugates (ADCs) that show encouraging anticancer activity in HER2-low patients. Their remarkable anticancer efficacy has sparked considerable interest and excitement in this developing subpopulation [6,7]. According to the findings of the Destiny-Breast04 research, trastuzumab deruxtecan was recently identified as a breakthrough drug by the FDA to treat HER2-low metastatic breast cancer [8].

In early breast cancer, the effectiveness of neoadjuvant chemotherapy (NAC) is evaluated based on pathological complete response (pCR) rates. pCR is a key therapeutic goal and has prognostic value [9]. There is limited information available regarding the rates of pCR in patients with HER2-low early breast cancer [10].

There is an ongoing debate about the impact of HER2-low expression on chemotherapy response and survival in patients at early stages [11]. Previous research suggests that HER2-low patients show limited predictive significance regarding pCR and survival following NAC [12]. However, a study by Denkert et al., which comprised four prospective neoadjuvant clinical trials, found that the HER2-low group differed significantly from the HER2-zero group, as they had a lower pCR rate but a higher survival rate [4]. Examining the impact of HER2 status (HER2-low vs. HER2-zero) on achieving pCR could help in tailoring neoadjuvant treatment options to improve results for this patient group [13]. Therefore, as this is still an unmet medical need, more study is necessary to understand how HER2 status affects pCR and survival [11].

Consequently, we performed a retrospective analysis to determine whether there are differences between HER2-low and HER2-zero breast cancer. This retrospective cohort study aims to examine the influence of HER2 status (HER2-low vs. HER2-zero) on the pathological response to neoadjuvant chemotherapy in breast cancer patients and to assess the HER2 status regarding survival outcomes in early-stage breast cancer.

## 2. Material and Methods

We retrospectively examined breast cancer patients treated with NAC from 2017 to 2023 in single cancer centers. The inclusion criteria for this study included the following: female gender, age older than 18, diagnosis of invasive BC, a HER2 immunohistochemistry (IHC) score of 0, 1+, or 2+/ISH negative, treatment with NAC followed by surgery with curative intent, and hormonal receptors that are either positive or negative. Patients with HER2-positive BC (IHC 3+ or IHC 2+ ISH amplified), clinical stage 4, with history of previous invasive breast carcinoma, or other primary neoplasia history, and without adequate data, were excluded.

Medical records were used to gather information on the following: age, menopausal status, histological subtype and grade, hormone receptor (HR) and HER2 status, Ki-67 expression, tumor localization, clinical T Stage, clinical N Stage, stage at diagnosis, type of surgery, post-operative pathological outcomes, and disease recurrence.

A pathologist independently evaluated HER2 and HR status on pretreatment biopsy specimens. Tumors with estrogen (ER) and/or progesterone receptor (PR) levels of ≥1% were considered HR-positive, while tumors with ER < 1% and PR < 1% were defined as triple-negative breast cancer (TNBC). HER2 status was assessed in accordance with the American Society of Clinical Oncology/College of American Pathologists (ASCO/CAP) guidelines [4]. A HER2-low status was defined by IHC+1 or +2/ISH non-amplified, and HER2-zero was determined by IHC 0.

Neoadjuvant chemotherapy agents included either conventionally scheduled or intensified dose-dense anthracyclines and taxanes for most patients, carboplatin for some patients with TNBC, and some other agents. None of the patients included in our analysis had received anti-HER2 therapy.

The study’s primary objective was to compare the pCR rates between the HER2-zero and HER2-low patient groups. Total pCR (tpCR) was defined as no residual invasive tumor cells in any resected specimens of the breast and axillary nodes (ypT0/is ypN0), stratified by HER2-zero and HER2-low. The second short-term efficacy endpoints included breast pCR (bpCR), defined as no residual invasive tumor cells in the breast (ypT0 yPN0/+), axillary lymph nodes pCR (npCR), defined as no residual invasive tumor cells in axillary lymph nodes (ypTany ypN0), and radiological CR (rCR), defined as no signs of disease in breast MRI (Magnetic Resonance Imaging).

Furthermore, the study compared the disease-free survival (DFS) of these two groups (HER2-low and HER2-zero). DFS was defined as the time from breast cancer diagnosis to the earliest locoregional or contralateral relapse, distant metastasis, or death from any cause. No-event patients were censored at the end of the last follow-up. Additional stratification was performed by HR status (HR-positive and TNBC).

The study was approved by the Ethics Committee of Istanbul Bakirkoy Dr. Sadi Konuk Training and Research Hospital (2024/24) and performed in accordance with the principles of the Declaration of Helsinki. Because of its retrospective nature, informed consent was waived.

### Statistical Analysis

Patients included in the study were grouped according to their HER2 status (HER2-low and HER2-zero). The ages of patients were represented by means and ranges. Other categorical clinical and pathologic variables were summarized and compared across groups using chi-square or Fisher’s exact test. Continuous variables were compared between the two groups using the Mann–Whitney U test. The differences in pathological response rates were evaluated as rate differences with 95% confidence intervals (CI) between patients with HER2-low and HER2-zero tumors. The effect level of pCR was investigated by univariate and multivariate logistic regression with 95% CI. Cox-regression (univariate and multivariate) and Kaplan–Meier were used in the survival analysis. A *p*-value of <0.05 was considered statistically significant. The SPSS 28.0 program was used for analyses.

## 3. Results

### 3.1. Patients and Tumor Characteristics

Overall, 134 patients were evaluated. The median follow-up was 23.0 months (95% CI 19.8–26.2). In total, 91 (67.9%) patients had the HER2-zero subtype, and 43 (32.1%) patients had the HER2-low subtype.

The baseline characteristics of the patients are described in Table 1. The median age was 48.9 ± 11.3. Menopause status was determined as 45.1% premenopausal, 42.9% postmenopausal, and 12.1% perimenopause in the HER2-zero group, while 62.8% premenopausal, 37.2% postmenopausal, and 0% perimenopause in the HER2-low group. There was a significant difference between the groups (*p* = 0.027). The proportion of premenopausal individuals in the HER2-low group was significantly higher. The predominant histological subtype in both groups was invasive ductal carcinoma, and both groups mainly had grade 3. The ratio of patients with Ki-67 > 20% was higher in both groups. No statistically significant difference was observed between the groups regarding histologic type and grade, Ki-67 index, clinical T stage, and clinical N stage (*p* = 0.056, *p* = 0.0.638, *p* = 0.088, *p* = 1.00, *p* = 0.295, respectively).

The molecular subgroup was 42.9% TNBC and 57.1% HR+ in the HER2-zero group, while 18.6% TNBC and 81.4% HR+ in the HER2-low group. There was a significant difference between the groups (*p* = 0.011). The HR+ ratio was significantly higher in the HER2-low group, while the TNBC ratio was lower than in the HER2-zero group.

In the HER2-zero group, 47.3% of the patients were stage 2 and 52.7% were stage 3; in the HER2-low group, 32.6% were stage 2 and 67.4% were stage 3. There was no statistical difference between the groups (*p* = 0.156). Tumor localization was the majority in the right breast in both groups (*p* = 0.741). Anthracyclines followed by taxanes were the preferred NACT regimen, 85.4% in HER2-low and 96.8% in HER2-zero subsets *p* = 0.892), with the highest proportion of dose-dense regimen being used in HER2-zero patients (43.9%) with no statistical value (0.580). Mastectomy and axillary dissection was performed for most patients in both groups (*p* = 0.999).

### 3.2. Predictive Value of HER2 Status on Pathological Response

Overall, pCR was achieved in 36 (26.9%) patients, with 24 (26.4%) in HER2-zero patients and 12 (27.9%) in HER2-low patients (*p* = 0.852). In univariate analysis, factors that could be associated with pCR, including age, menopausal status, histology, grade, tumor location, cT stage, cN stage, stage at diagnosis, Ki-67 index, HER2, HR status, chemotherapy types, and a present dose-dense regimen were tested. The cN stage, Ki-67 index, and HR status were significantly associated with pCR (*p* = 0.021, *p* = 0.002, *p* < 0.001, respectively) (Table 2). However, the cN stage and Ki-67 index lost significance in multivariate analysis. The only variable that remained significant in relation to pCR was the HR status (*p* = 0.002).

**Figure 1:**
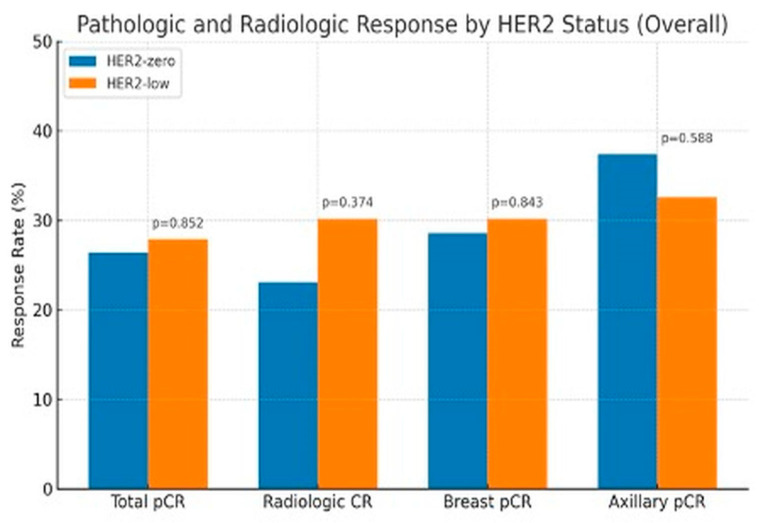
Pathologic and Radiologic Response by HER2 status.

No significant differences in pCR rates were seen in HER2-zero and HER2-low groups, including the rates of tpCR (26.4% vs. 27.9%, *p* = 0.852), bpCR (28.6% vs. 30.2%, *p* = 0.843), npCR (37.4% vs. 32.6%, *p* = 0.588), and rCR (23.1% vs. 30.2%, *p* = 0.374) (Figure 1).

**Figure 2:**
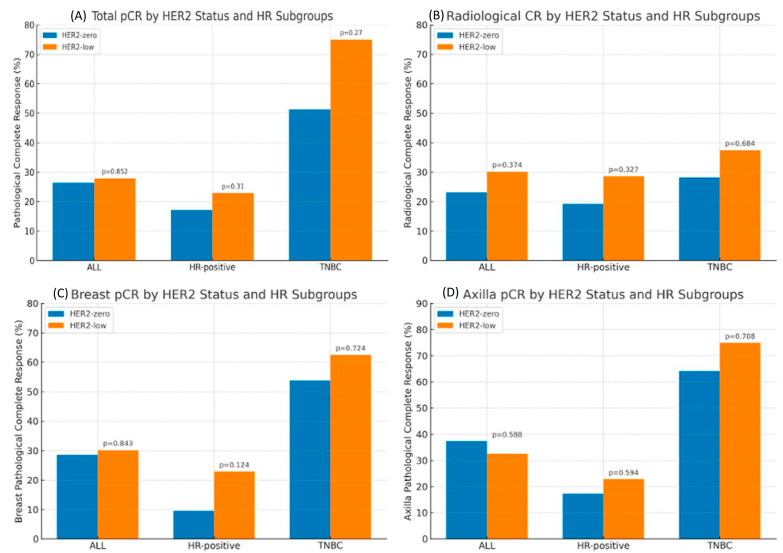
Total pCR (**A**), Radiologic CR (**B**), Breast pCR (**C**) and Axilla pCR (**D**) by HER2 Status and HR Subgroups.

In the subgroup analysis according to HR status, HER2 status was not associated with the rates of pCR (tpCR, rCR, bpCR, and npCR) in patients with the HR-positive subgroup in logistic regression analysis (all *p* > 0.05). Similar results were seen in the TNBC subgroup; HER2 status was not associated with the rates of pCR (tpCR, rCR, bpCR, and npCR) (all *p* > 0.05) (Figure 2).

### 3.3. Prognostic Value of HER2 Status on Patient Survival

The median DFS is 69.6 months (95% CI 61.4–77.7). In univariate analysis, factors that could be associated with DFS, including age, menopausal status, histology, grade, tumor location, cT stage, cN stage, stage at diagnosis, Ki-67 index, HER2 status, HR status, chemotherapy types, a present dose-dense regimen, and pCR rates, were tested. The grade, tpCR, bpCR, and npCR were significantly associated with DFS (*p* = 0.002, *p* = 0.023, *p* = 0.018, *p* = 0.005, respectively). However, in multivariate analysis, these were not statistically significant (Table 3).

**Figure 3:**
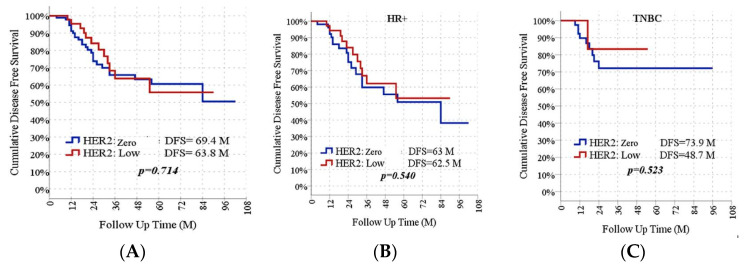
DFS Rates According to HER2 Status in All Group (**A**), HR-Positive Subgroup (**B**), and TNBC Subgroups (**C**).

The median survival time of HER2-zero individuals was 69.4 months (95% CI 59.6–79.2). In HER2-low individuals, the median survival time was 63.8 months (95% CI 52.0–75.6). There was no significant difference in survival between the groups (*p* = 0.714). Survival outcomes were further analyzed according to HR status. In the HR-positive subgroup, the DFS of HER2-zero patients was similar to their HER2-low counterparts (63.0 vs. 62.5 months, *p* = 0.540). In the TNBC subgroups, there was no statistical difference in the DFS between HER2-zero patients and their HER2-low counterparts (73.9 vs. 48.7 months, *p* = 0.523) (Figure 3).

## 4. Discussion

In clinical research and practice guidelines, the HER2-low category has received a lot of attention lately. Based on prognosis, responsiveness to treatment, and molecular features, it is suggested that HER2-low breast cancer could be identified as a separate subtype [14]. The European Society for Medical Oncology (ESMO) released an expert consensus statement in 2023 regarding the diagnosis, treatment, and definition of HER2-low breast cancer [15]. Clinical trial data indicates that antibody–drug conjugates could achieve outcomes in treating cancers with low to moderate HER2 expression [16]. Despite the data obtained from studies on metastatic disease, it is not known how the HER2-low group differs in terms of treatment response and prognosis in locally advanced disease.

Patients in this cohort were classified as HER2-low at a rate of 32.1%, a slightly lower frequency than observed in previous reports, in which the prevalence of this subtype ranged from 40 to 50 [17,18]. One reason could be that, until recently, there were no clinical implications for the difference between HER2 0 and HER2 +1, which could affect pathologists‘ necessity to assess low scores accurately. Moreover, interobserver concordance for HER2 IHC scoring is mixed. Some studies demonstrated significant discordance, particularly in distinguishing between IHC 2+ and IHC 0/1+ scores [19,20]. Therefore, tumors that were in fact HER2 +1 may have been reported as HER2 negative, which may have affected the results in our cohort.

Studies conducted on the effect of low HER2 status on pathological responses following NAC have produced different results [14]. According to a retrospective analysis of 331 patients who presented at San Antonio 2020, patients with HER2-low tumors had a considerably worse pCR compared to those with HER-zero tumors in the overall cohort. However, no differences were observed in subgroups based on HR status [21]. Some studies did not support HER2-low as a biologically unique subtype of breast cancer [13,22]. Some studies have implied that the HER2-low status harbors more aggressive biological characteristics (such as grade, cN stage, and Ki-67 index), which may explain the differentiation in pCR [23]. Our research, however, did not confirm the view that HER2-low and HER2-zero breast cancers vary biologically. There were no differences between the two groups regarding tumor characteristics, and these findings may have contributed to similar results for pCRs.

One study found that having a HER2-low status did not affect the response rate following NAC [13]. A retrospective trial of 449 TNBC patients showed similar findings. There was no significant difference in the pCR rate between the HER2-low and HER2-zero groups (35.7% vs. 41.8%, *p* = 0.284) [24]. A comprehensive analysis of 331 patients revealed no significant difference in the pCR rates between the hormone receptor-positive HER2-zero and HER2-low subgroups, which showed rates of 8% and 13%, respectively [21]. Our study reported that HER2-low tumors had a similar pCR rate to HER2-zero tumors. Similar to those studies in the literature, no differences in pCR rates were determined in the subgroup analysis according to HR status. Moreover, we performed a more detailed analysis to better understand how a HER2-low status may affect the difference in pCR. We found that both groups had similar tpCR, rCR, bpCR, and npCR rates.

When compared to the individuals with HER2-zero levels, breast cancer with low HER2 levels exhibited an essential rise in the HR-positive rate (92.31% vs. 70.39%, *p* < 0.001) [24]. In our patient population, the HR positivity rate was significantly higher in the HER2-low patient group than in the HER2-zero group (81.4% vs. 57.1%, *p* = 0.011). According to recent studies, luminal-related genes are more heavily present in HER2-low breast malignancies than in HER2-zero tumors [25]. Activating the PI3K/AKT pathway could be a critical marker for predicting a lack of response to NAC in patients with breast cancer [26]. Genetic differences that lead to tumor heterogeneity among the included patients may be one factor contributing to changes in study results. Moreover, further genetic studies are required to understand the connection between treatment outcomes and HER2-low expression.

There is controversy on the prognosis of HER2-low breast cancer currently. We analyzed the DFS in HER2-low and HER2-zero entities in the overall population and the HR-positive/TNBC subgroups. However, no significant differences in DFS were detected between patients with HER2-low or HER2-zero tumors. The outcomes were consistent with a study by Xu et al. [24]. In another group of 3,689 patients, the majority of whom had advanced BC, a HER2-low status did not have a prognostic effect on OS [25]. According to different multicenter research, in non-metastatic patients, HER2-low breast cancer had improved OS and relapse-free survival (RFS) [27]. In one study, DFS for the entire group showed statistical significance, but when HR-positive and triple-negative breast cancer tumors were analyzed separately, no statistical significance was detected [28]. Our results indicated that the DFS was similar in both groups in HR-positive and TNBC patients (*p* = 0.540, *p* = 0.523, respectively).

Several limitations existed in the current study. First, it was a retrospective study, which may have been responsible for both selection and information bias. Second, it was a single-center cohort study. Third, the sample size was relatively small. In addition, the IHC assessment of HER2 lacked the evaluation of a single pathologist, and results might have varied from person to person. Fourth, we could not calculate OS when evaluating survival outcomes because our follow-up time was short; longer follow-ups are needed.

## 5. Conclusions

In conclusion, our results showed that HER2-low BC did not have a distinct biology, NACT responses, or prognosis; even so, it may reflect a specially interesting population in which to test new HER2-targeting agents. Therefore, efforts should be made to better identify those patients in real-life settings. Further research is warranted because HER2-low patients may represent a distinct subset of patients for investigating novel therapeutic approaches to improve BC outcomes.

## Figures and Tables

**Table 1:** Clinicopathological Characteristics According to HER2 Status.

		HER2			*p*
		HER2-Zero	HER2-Low	Total	
		*n* (%)	*n* (%)	*n* (%)	
**Age (Mean ± SD) Median**		**(49.75 ± 11.11) 48**	**(46.95 ± 11.6) 44**	**(48.85 ± 11.31) 47**	**0.183**
Menopausal Status	Premenopausal	41 (45.1)	27 (62.8)	68 (50.7)	0.027
	Postmenopausal	39 (42.9)	16 (37.2)	55 (41.0)	
	Perimenopausal	11 (12.1)	0 (0.0)	11 (8.2)	
Histology	Invasive Duktal	83 (91.2)	33 (76.7)	116 (86.6)	0.056
	Invasive Lobuler	5 (5.5)	5 (11.6)	10 (7.5)	
	Other	3 (3.3)	5 (11.6)	8 (6.0)	
Histological Grade	1–2	35 (38.5)	14 (32.6)	49 (36.6)	0.638
	3	56 (61.5)	29 (67.4)	85 (63.4)	
Ki-67 (%)	≤20%	21 (23.1)	16 (37.2)	37 (27.6)	0.088
	>20%	70 (76.9)	27 (62.8)	97 (72.4)	
Subgroup	TNBC	39 (42.9)	8 (18.6)	47 (35.1)	0.011
	HR+	52 (57.1)	35 (81.4)	87 (64.9)	
Stage at Diagnosis	Stage 2	43 (47.3)	14 (32.6)	57 (42.5)	0.156
	Stage 3	48 (52.7)	29 (67.4)	77 (57.5)	
Tumor Localization	Right Breast	44 (48.4)	19 (44.2)	63 (47.0)	0.741
	Left Breast	46 (50.5)	23 (53.5)	69 (51.5)	
	Bilateral	1 (1.1)	1 (2.3)	2 (1.5)	
Clinical T Stage	1	5 (5.5)	2 (4.7)	7 (5.2)	1.000
	2	55 (60.4)	26 (60.5)	81 (60.4)	
	3	26 (28.6)	13 (30.2)	39 (29.1)	
	4	5 (5.5)	2 (4.7)	7 (5.2)	
Clinical N Stage	0	9 (9.9)	4 (9.3)	13 (9.7)	0.295
	1	48 (52.7)	17 (39.5)	65 (48.5)	
	2	29 (31.9)	21 (48.8)	50 (37.3)	
	3	5 (5.5)	1 (2.3)	6 (4.5)	
Neoadjuvant CT regimen	Antracycline +/− taxane No antracycline or taxane	87 (95.7) 4 (4.3)	41 (95.4) 2 (4.6)	128 (95.6) 6 (4.4)	0.892
Dose-dense regimen	No Yes	51 (56.1) 40 (43.9)	29 (67.5) 14 (32.5)	80 (59.7) 54 (40.3)	0.580
Surgical Type	Mastectomy+ALND (MRM)	31 (34.1)	19 (44.2)	50 (37.3)	0.999
	Mastectomy+SLNB	11 (12.1)	6 (14.0)	17 (12.7)	
	BCS+ALND	22 (24.2)	11 (25.6)	33 (24.6)	
	BCS+SLNB	27 (29.7)	7 (16.3)	34 (25.4)	

Abbreviations: HER2: Human Epidermal Growth Factor Receptor 2. TNBC: Triple-Negative Breast Cancer. HR: Hormone Receptor. CT: Chemotherapy. ALND: Axillary Lymph Node Dissection. BCS: Breast-conserving Surgery. MRM: Modified Radical Mastectomy. SLNB: Sentinel Lymph Node Biopsy.

**Table 2:** pCR Analysis by Clinicopathological Parameters.

		Total pCR (−) (*n*:98)		Total pCR (+) (*n*:36)		*p*	
		Mean ± SD/*n*−%	Median	Mean ± SD/*n*−%	Median		
***Age***		49.7 ± 11.7	48.0	46.6 ± 10.0	44.5	0.164	^m^
***Menopausal Status***							
Premenopausal		47	48.0	21	58.3	0.304	^X2^
Postmenopausal		44	44.9	11	30.6		
Perimenopausal		7	7.1	4	11.1		
***Histology***							
Invasive Duktal		84	85.7	32	88.9	0.867	^X2^
Invasive Lobuler		8	8.2	2	5.6		
Other		6	6.1	2	5.6		
***Histological Grade***	I	2	2.0	0	0.0	0.638	^X2^
	II	35	35.7	12	33.3		
	III	61	62.2	24	66.7		
***Ki-67*** (%) ≤ 20%		33	89.1	4	10.9	***0.0002***	^m^
> 20%		65	67.0	32	33.0		
***HER2 status***	Zero	67	68.4	24	66.7	0.852	^X2^
	Low	31	31.6	12	33.3		
***Subgroup***	TNBC	21	21.4	26	72.2	***<0.001***	^X2^
	HR+	77	78.6	10	27.8		
***Clinical T Stage***	I	4	4.1	3	8.3	0.577	^X2^
	II	59	60.2	22	61.1		
	III	29	29.6	10	27.8		
	IV	6	6.1	1	2.8		
***Clinical N Stage***	0	6	6.1	7	19.4	***0.021***	^X2^
	I	48	49.0	17	47.2		
	II	38	38.8	12	33.3		
	III	6	6.1	0	0.0		
***Stage at Diagnosis***	II	37	37.8	20	55.6	0.065	^X2^
	III	61	62.2	16	44.4		
***Neoadjuvant CT regimen***	Antracycline +/− taxane	95	95.9	33	94.2	0.867	
	No antracycline or taxane	4	4.1	2	5.8		
***Dose-dense regimen***	No	61	61.6	19	54.2	0.654	
	Yes	38	38.4	16	45.8		
***Tumor Localization***	Right Breast	45	46.9	18	50.0	0.749	^X2^
	Left Breast	51	53.1	18	50.0		

^m^ Mann–Whitney u test/^X²^ Ki-kare test. Abbreviations: HER2: Human Epidermal Growth Factor Receptor 2. TNBC: Triple-Negative Breast Cancer. HR: Hormone Receptor. CT: Chemotherapy. pCR: Pathological Complete Response.

**Table 3:** Univariate Analysis of DFS.

	Univariate			
	HR	95% CI		*p*
Age	1.008	0.981	1.036	0.575
Menopausal Status	1.322	0.828	2.109	0.242
Histology	1.215	0.738	2.000	0.443
Histological Grade	3.362	1.565	7.222	***0.002***
Ki-67%	0.994	0.981	1.007	0.350
HER2 status	0.881	0.445	1.744	0.715
Subgroup (TNBC/HR+)	1.496	0.728	3.072	0.273
cT Stage	1.369	0.868	2.157	0.176
cN Stage	1.441	0.940	2.210	0.094
Stage at Diagnosis	1.838	0.941	3.589	0.075
Tumor Localization	0.884	0.492	1.589	0.681
Neoadjuvant CT regimen	1.298	0.734	2.100	0.712
Dose-dense regimen	1.354	0.811	2.109	0.542
Total pCR	0.302	0.107	0.849	***0.023***
Radiological CR	0.785	0.361	1.709	0.543
Breast pCR	0.320	0.125	0.821	***0.018***
Axilla pCR	0.290	0.121	0.694	***0.005***

Cox Regresyon (Forward LR). Abbreviations: HER2: Human Epidermal Growth Factor Receptor 2. TNBC: Triple-Negative Breast Cancer. HR: Hormone Receptor. CT: Chemotherapy. pCR: Pathological Complete Response.

## Data Availability

The data sets generated during and/or analyzed during the current study are available from the corresponding author on reasonable request.
